# The jumping spider *Saitis barbipes* lacks a red photoreceptor to see its own sexually dimorphic red coloration

**DOI:** 10.1007/s00114-021-01774-6

**Published:** 2021-12-11

**Authors:** Mateusz Glenszczyk, David Outomuro, Matjaž Gregorič, Simona Kralj-Fišer, Jutta M. Schneider, Dan-Eric Nilsson, Nathan I. Morehouse, Cynthia Tedore

**Affiliations:** 1 Zoological Institute, University of Hamburg, Martin-Luther-King Platz 3, 20146 Hamburg, Germany; 2 Institute of Biology, Biotechnology and Environmental Protection, University of Silesia, Bankowa 9, 40-007 Katowice, Poland; 3 Department of Biological Sciences, University of Cincinnati, Cincinnati, OH USA; 4 Research Centre of the Slovenian Academy of Sciences and Arts, Jovan Hadži Institute of Biology, Novi trg 2, Ljubljana, Slovenia; 5 Lund Vision Group, Lund University, Sölvegatan 35, 223 62 Lund, Sweden

**Keywords:** Color vision, Visual signaling, Salticidae, Sexual selection, Computational filters

## Abstract

Examining the role of color in mate choice without testing what colors the study animal is capable of seeing can lead to ill-posed hypotheses and erroneous conclusions. Here, we test the seemingly reasonable assumption that the sexually dimorphic red coloration of the male jumping spider *Saitis barbipes* is distinguishable, by females, from adjacent black color patches. Using microspectrophotometry, we find clear evidence for photoreceptor classes with maximal sensitivity in the UV (359 nm) and green (526 nm), inconclusive evidence for a photoreceptor maximally sensitive in the blue (451 nm), and no evidence for a red photoreceptor. No colored filters within the lens or retina could be found to shift green sensitivity to red. To quantify and visualize whether females may nevertheless be capable of discriminating red from black color patches, we take multispectral images of males and calculate photoreceptor excitations and color contrasts between color patches. Red patches would be, at best, barely discriminable from black, and not discriminable from a low-luminance green. Some color patches that appear achromatic to human eyes, such as beige and white, strongly absorb UV wavelengths and would appear as brighter “spider-greens” to *S. barbipes* than the red color patches. Unexpectedly, we discover an iridescent UV patch that contrasts strongly with the UV-absorbing surfaces dominating the rest of the spider. We propose that red and black coloration may serve identical purposes in sexual signaling, functioning to generate strong achromatic contrast with the visual background. The potential functional significance of red coloration outside of sexual signaling is discussed.

**Supplementary Information:**

The online version contains supplementary material available at 10.1007/s00114-021-01774-6.

## Introduction

When generating hypotheses as to the functional significance of animal color patterns, we humans cannot help but be biased by colors and contrasts that are conspicuous to our own eyes. Yet the human color vision system, with no UV photoreceptor class but separate green and red photoreceptor classes, is rather unusual outside of primates, being representative of few animal taxa. In spite of this, the functional significance of many animal color patterns has been investigated without knowledge of the spectral positions of their photoreceptors, with hypotheses and interpretations of data guided largely by what we see with our own eyes and/or by the visual systems of related taxa (Baird et al. 2013; Guillermo-Ferreira et al. 2014; Lovari et al. 2015; Portik et al. 2019; Greener et al. 2020; Johnson et al. 2020; Butterworth et al. 2021).

In the absence of photoreceptor spectral sensitivity data, color pattern reflectance spectra provide a perceptually unbiased measurement of animal colors. However, reflectance spectra can be misleading in other ways, as they may cover a broader portion of the spectrum than the animal is sensitive to. Moreover, reflectance spectra contain finer spectral resolution (usually 1–5 nm resolution) than animal eyes can resolve, which utilize opsin-based visual pigments with spectrally overlapping sensitivities, each of which spans 100–400 nm (depending on spectral position and the presence of filtering pigments). Color differences revealed by the fine and discrete sampling resolution of a spectrometer may not always be perceptible to animals due to the coarse spectral resolution provided by a limited set of differentially sensitive photoreceptor classes.

Testing functional hypotheses about animal coloration without knowledge of animal spectral sensitivities has become especially common in the jumping spider (Salticidae) literature over the past 15 years; see, for example, studies of *Siler semiglaucus* (Zhou et al. 2021), *Evarcha culicivora* (Cross et al. 2020), *Maratus volans* (Girard et al. 2018), *Habronattus pyrrithrix* (Taylor and McGraw 2013), *Lyssomanes viridis* (Tedore and Johnsen 2012), *Phintella vittata* (Li et al. 2008), and *Cosmophasis umbratica* (Lim et al. 2008). In general, jumping spiders’ sexually dimorphic color patterns, dynamic courtship dances, and high-acuity vision make them excellent models for testing hypotheses about sexual selection (Harland et al. 2012). Without basic knowledge about what colors they can see, however, our ability to interpret data collected about, for example, the condition dependence of coloration, species and sex recognition, and mate choice is limited. Zurek et al. (2015) took steps to remedy this situation by measuring the spectral sensitivities of *Habronattus pyrrithrix*, an emerging model for sexual signaling research. *H. pyrrithrix* was found to have two visual pigments with peak sensitivity in the ultraviolet (UV) and green spectral regions, in separate photoreceptor classes, as well as a third photoreceptor class expressing the green visual pigment but with incoming light filtered by a photostable red pigment, effectively shifting its peak spectral sensitivity into the red. This means that hypotheses about red coloration previously tested in this species were well-posed (Taylor and McGraw 2013; Taylor et al. 2014, 2016). Whether the same can be said for studies of other species’ coloration remains to be determined. The few other salticid taxa whose spectral sensitivities have been characterized have not been utilized in studies of sexually dimorphic coloration. Electrophysiological recordings have provided clear evidence of UV- and green-sensitive photoreceptors in *Phidippus regius* and *Servaea vestita* (De Voe 1975; Blest et al. 1981), and, due to small sample size, what can be considered preliminary evidence for UV-, blue-, green-, and yellow-sensitive photoreceptors in *Menemerus confusus* (Yamashita and Tateda 1976). More recently, immunofluorescence staining has shown *Hasarius adansoni* to express only ultraviolet and green opsins in the retina (Nagata et al. 2012).

Here, we seek to reverse the trend of testing functional hypotheses about salticid color patterns in the absence of basic physiological data on their vision. We do this by beginning an investigation of the colorful salticid *Saitis barbipes* with a microspectrophotometric investigation of its color vision system. We also check for the presence of a red filter in the retina, as was found in *H. pyrrithrix*, and in the lens, by measuring lens transmittance. We then use this information to generate computational filters mimicking *S. barbipes* effective spectral sensitivities using a multispectral camera equipped with seven filters, following Tedore and Nilsson (2021). This technique allows us to visualize and quantify how the male color pattern appears through female eyes. With this information, we are well-equipped to develop and test hypotheses as to the function of male *S. barbipes* coloration in future studies.

*S. barbipes* is a promising species for sexual signaling research for several reasons. *S. barbipes* are highly sexually dimorphic, with males exhibiting an orange band above the eyes and red and black patches on the third pair of legs, which are waved vigorously during courtship dances (Wearing et al. 2014). Importantly, when females of this species are disinterested in a male, they often appear to signal rejection by raising the abdomen (MG & CT, pers. obs.), similar to what has previously been reported in the closely related *Maratus* genus (Girard et al. 2015). This apparent rejection signal is unusual among salticids and opens up the possibility of scoring female receptivity before copulation takes place, including in response to video playbacks and computer animations. Additionally, *S. barbipes* is widely distributed in southern Europe and can be collected in large enough numbers for behavioral research.

## Methods

### Spider collection

Thirty male and seven female *Saitis barbipes* were collected as subadults and adults from a submediterranean forest in Osp, Slovenia (45°34′47.7″N 13°51′20.0″E), in June 2019 and May 2021. This forest community is classified as *Aristolochio luteae-Quercetum pubescentis* (Poldini 2008), occurs on calcareous bedrock, and is composed of mixed stands of coppice and seed source trees, with a high tree level and well-developed shrub and herb layers (Marinček & Čarni 2002). Most spiders were encountered in partial shade at the forest edge, in the herb layer among leaf litter and large rocks. The spiders were spotted by eye and collected using a simplified aspirator. After species identification was verified in the laboratory of ZRC SAZU, Slovenia, thirty-one spiders were transferred to the University of Hamburg, Germany, for lens transmittance measurements (*N* = 3 males and 3 females) and multispectral imaging (*N* = 24 males and 1 female), and the remaining spiders were transferred to the University of Cincinnati, USA, for microspectrophotometry (*N* = 3 males and 3 females).

### Microspectrophotometry

Microspectrophotometry (MSP) was used on cryosections of the principal eye retinas of *S. barbipes* to measure photoreceptor absorbance profiles. Spiders were dark-adapted overnight before cryosectioning. Sample preparation, cryosectioning, and MSP measurement occurred under dim red light to avoid bleaching of retinal tissues. The legs and opisthosoma of the spider were cut off and the cephalothorax was flash-frozen in Tissue Plus OCT Compound (Fisher Healthcare, Houston, Texas). The embedded cephalothoraxes were cryosectioned in the coronal plane using a thickness of 13 μm on a Leica CM1860 cryostat at − 20 °C. All sections that contained the principal eye retinal tissue were kept and inspected in the MSP. Prior to measuring, sections were placed between two glass cover slips (22 × 22 − 1 Fisherfinest, Fisher Scientific, Pittsburgh, Pennsylvania) and immersed in mineral oil (Fisher Scientific, Fair Lawn, New Jersey) surrounded by a ring of silicone grease (Dow Corning Corporation, Midland, Michigan).

The absorbance of individual photoreceptor cells was measured between 300 and 700 nm using a custom-built single beam, scanning MSP with a 32 × Ultrafluar objective and a 32 × Ultrafluar condenser (Carl Zeiss, Germany). The light source was a xenon arc lamp (XBO 75 W/2, Osram Sylvania, Wilmington, MA) and it was dispersed from 300 to 700 nm in steps of 1 nm using a monochromator (H10 UV, Jobin Yvon Instruments, SA, Edison, NJ). First, a reference scan was measured in a clear area away from the section but within the mineral oil and subsequently subtracted from measurements to account for light absorption by the preparation itself (e.g., mineral oil and coverslips). Second, measurements were performed in areas with photoreceptor cells following this procedure: a photoreceptor was measured, then photobleached for 30 s using white light, and then re-measured. The difference between the pre-bleach spectrum and the photobleached spectrum was used to confirm the presence of photopigments. The peak sensitivities of the photoreceptors were then estimated by fitting pre-bleach absorbance curves to visual templates (Govardovskii et al. 2000) representing a range of possible values for the wavelength of peak sensitivity (i.e., alpha-peak lambda max values; see Govardovskii et al. 2000), with the best fit template (and associated lambda max) identified using least-squares model comparisons. On rare occasions, noise in the short wavelength region of the absorbance spectra (i.e., where the MSP signal-to-noise ratio is lowest) required manual adjustment to reduce the impact of this noise on model estimations. After being measured in the MSP, each section was visually inspected for the presence of possible intraretinal filters (as have been found in *H. pyrrithrix*; see Zurek et al., 2015) under a Leica ICC50 HD microscope using a 40 × HI-Plan objective and bright light.

### Lens transmittance

Spiders were killed by over-anesthesia with CO_2_. Each principal eye lens was excised and rinsed in spider Ringer’s solution (190 NaCl, 2 KCl, 4 MgCl_2_, 4 CaCl_2_, and 1 Na_2_HPO_4_ (units in mmol/l) (Schartau and Leidescher 1983)) and kept in this solution until measurement. The lens consisted of a rigid cornea contiguous with the exoskeleton, and a softer, internal lens bathed in vitreous fluid. In intact spiders, these two optical components were attached to one another at the point at which the corneal edge meets the exoskeleton. This attachment was preserved during lens excision such that the transmittance of both optical components was measured together. For each spider, the entire process, from the beginning of dissection to the measurement of both principal eye lenses, was completed within about 1 h. One male lens was accidentally punctured during excision and was not measured.

The illumination light path consisted of a pulsed-xenon light source (PX-2) directed through a 115 µm extreme solarization-resistant optical fiber (QP115-2-XSR) and collimating lens (74-UV). The spider lens was placed in the center of the light path on a custom-built lens holder, which consisted of a 0.5-mm-thick sheet of black plastic with a 100 µm hole drilled through it. The upper part of the hole was widened and cup-shaped to cradle the lens over the 100 µm aperture in such a way that only light passing through the lens as it would in a living spider made it through the lens and into the measurement path. A small drop of spider Ringer’s solution was placed in the hole such that the part of the lens that would normally be bathed in vitreous fluid was sitting in solution, whereas the air-facing portion was dry. The measurement path consisted of a collimating lens (74-UV) connected to a 1000 µm solarization-resistant optical fiber (QP1000-2-SR), which directed the light to a spectrometer (QEPRO) (Suppl. Figure 1). All optical fibers, collimating lenses, and the spectrometer were sourced from Ocean Optics (Ostfildern, Germany). The placement of the lens in its holder and its alignment with the beam path was checked through an obliquely mounted microscope. The spider lens was measured first, followed by a reference measurement with the lens and Ringer’s solution removed from the plastic lens mount. Transmittance was calculated by dividing the first measurement by the second.

### Multispectral imaging and visual modeling

Each spider was killed by placing it in an Eppendorf tube in a − 80 °C freezer. Such flash freezing did not visibly affect the appearance of spider colors and has previously been found not to affect the chroma and brightness of the red hair and cuticle of the salticid *Lyssomanes viridis* (CT, unpublished data). Shortly before the spider was to be photographed, it was removed from the freezer and the ventral surface of its prosoma was glued to the head of a nail. The nail was stuck into a flat piece of styrofoam covered with undyed brown paper having a reflectance spectrum similar to that of leaf litter. The spider’s ornamented third pair of legs were posed in a display position using bent insect pins. For photography, the mounted spider was placed in front of a 20% reflective 2 inch fluorilon gray standard (Avian Technologies, New London, NH, USA), which reflects light evenly across the UV–VIS spectrum.

**Fig. 1:**
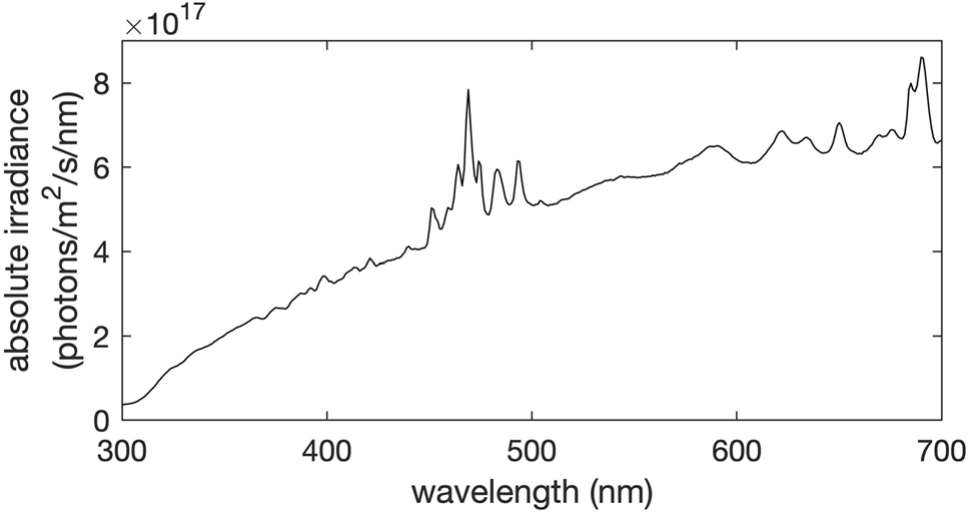
Absolute irradiance of the xenon light source (Seric XE-140BF) used to illuminate specimens for multispectral imaging

Images were taken in a dark room under a xenon light source (XE-140BF, Seric Ltd., Tokyo, Japan) closely mimicking the spectrum of natural daylight. The lamp’s irradiance spectrum was measured with a spectrophotometer (QE Pro) fitted with an extreme solarization-resistant (XSR) fiber optic cable and cosine corrector (CC-3-UV) that had been calibrated to absolute light intensities using a factory-calibrated deuterium and tungsten halogen light source (DH-3P-CAL), all sourced from Ocean Optics (Ostfildern, Germany) (Fig. 1). The lamp was tilted 60° from vertical and the spider was placed in the center of the cone of light emanating from it.

**Fig. 2:**
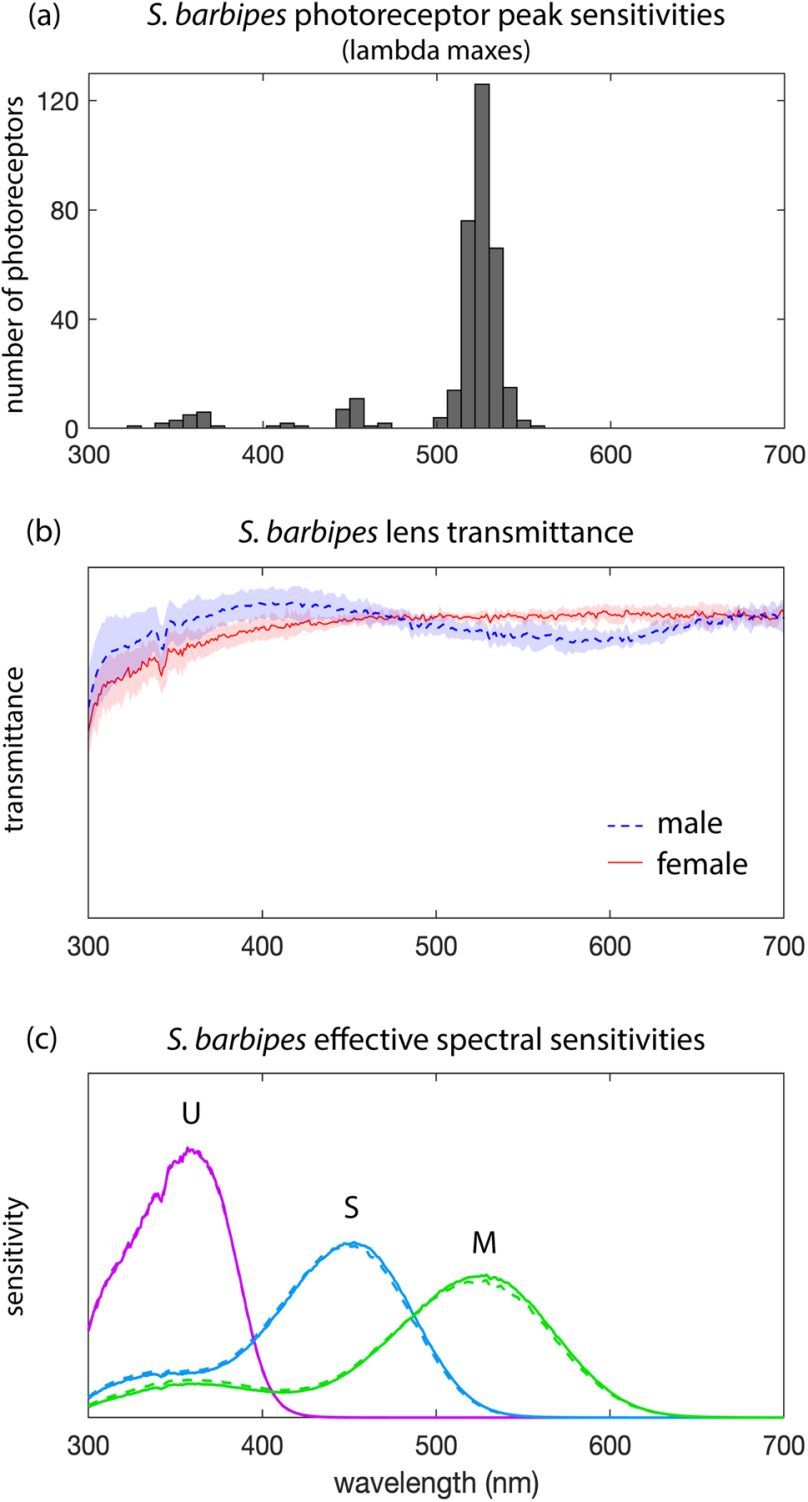
Spectral properties of *S. barbipes* eyes. **a** Frequency histogram of *S. barbipes* photoreceptors’ peak spectral sensitivities. Note that the blue photoreceptor may in fact be the inactive metarhodopsin state of the UV photoreceptor and would therefore not contribute to color discrimination. **b **Mean ± standard deviation of area-normalized lens transmittance spectra from the principal eyes of *S. barbipes* males (5 lenses from 3 individuals) and females (6 lenses from 3 individuals). **c** Area-normalized effective spectral sensitivities of male (dashed) and female (solid) photoreceptors generated by the product of lens transmittance and opsin absorption spectra generated by the template of Govardovskii et al. (2000). U, S, and M stand for ultraviolet-, short-, and medium-wavelength sensitivity, respectively

**Fig. 3:**
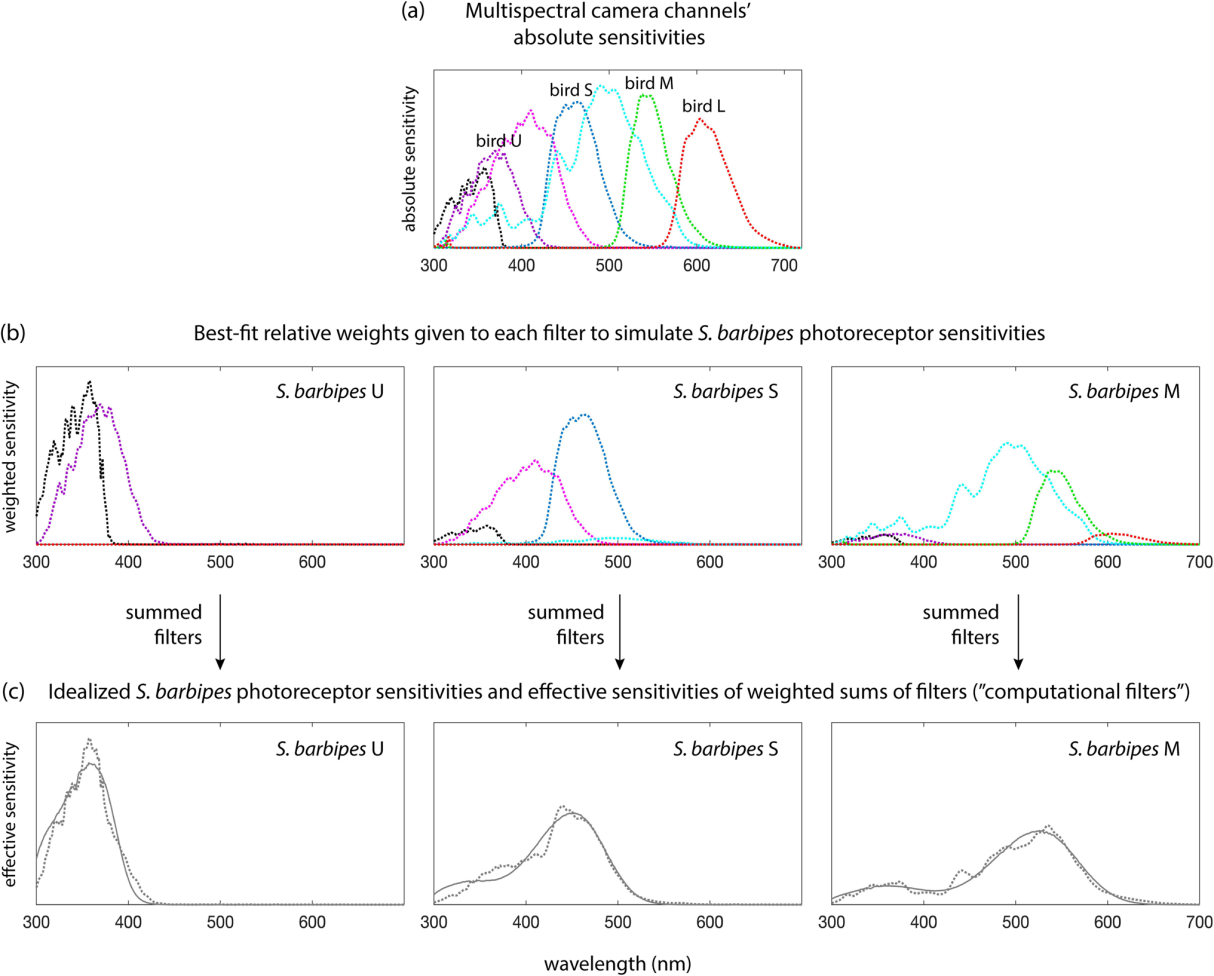
Pictorial explanation of computational filter generation. **a** Multispectral camera channels’ absolute spectral sensitivities in arbitrary units; labeled channels indicate those used to simulate avian vision. **b** Best-fit relative weights given to each filter in (a) to generate the desired *S. barbipes* spectral sensitivities shown in (c). **c** Summed weighted filters generate “computational filters” (dotted) that mimic idealized *S. barbipes* spectral sensitivities (solid) (all area-normalized). U, S, M, and L stand for ultraviolet-, short-, medium-, and long-wavelength sensitivity, respectively

Images were taken using the multispectral camera and five of the bird-based optical filters described in Tedore and Nilsson (2019). Two new filters were added to the system, which enabled the use of the computational filter technique described in Tedore and Nilsson (2021) to closely mimic *S. barbipes* spectral sensitivities (Figs. 2 and 3). This technique takes a weighted sum of pre-existing camera filters to generate new spectral sensitivities. First, the spectral sensitivity of each real camera channel *i* was calculated as:

1 $${{F}_{i}(\lambda )=S}_{\mathrm{sensor}}(\lambda ){ T}_{\mathrm{lens}}(\lambda ) {T}_{\mathrm{IRblock}}(\lambda ) {T}_{\mathrm{filter},i}(\lambda )$$

where *S*_sensor_(λ) is the spectral sensitivity of the camera sensor, *T*_lens_(λ) is the transmittance spectrum of the camera lens, *T*_IRblock_(λ) is the transmittance spectrum of an infrared blocking filter mounted on the front of the lens, and *T*_filter,*i*_(λ) is the transmittance spectrum of camera filter *i*. Next, we used constrained linear least squares to solve for a set of seven nonnegative coefficients to multiply by each camera channel such that the sum of the seven channels would generate a spectral shape matching each of *S. barbipes*’ spectral sensitivity curves. Such computational filters were generated by solving for the set of seven coefficients that best satisfies the following equation, while constraining the solution to prevent negative coefficients:

2 $$R_j(\lambda)T_{\mathrm l}(\lambda)=aF_1(\lambda)+bF_2(\lambda)+{cF}_3(\lambda)+{dF}_4(\lambda)+{eF}_5(\lambda)+{fF}_6(\lambda)+{gF}_7(\lambda),$$

where *R*_*j*_(λ) is the spectral sensitivity of photoreceptor *j* (generated by the template of (Govardovskii et al. 2000)), and *T*_l_(λ) is the transmittance spectrum of the *Saitis barbipes* lens.

**Fig. 4:**
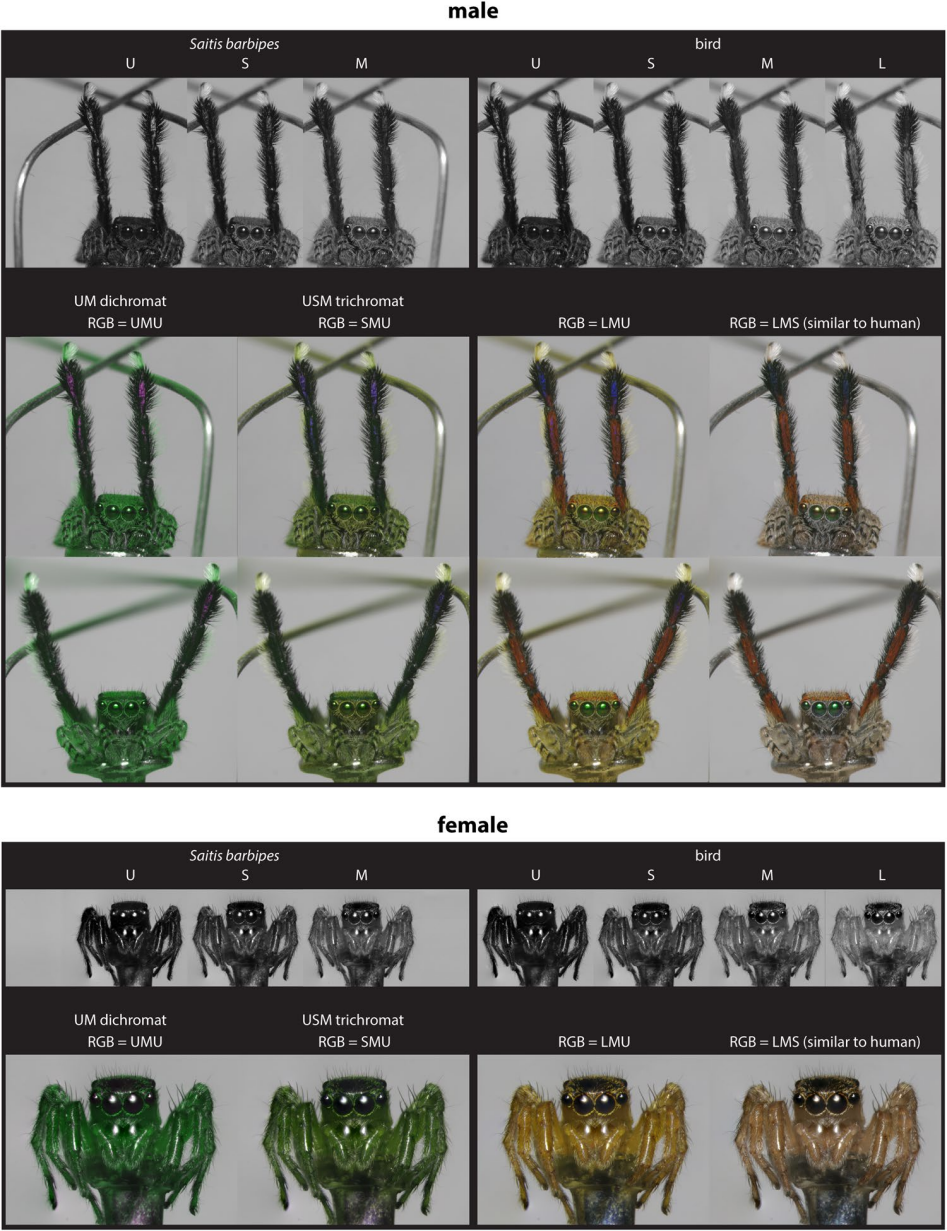
Receptor excitation images of *S. barbipes* males (upper panel) and females (lower panel) as seen by their own visual system (left) and birds (right) for comparison. Grayscale images represent the receptor excitation values of individual photoreceptor classes, with dark pixels corresponding to low receptor excitation and light pixels to high receptor excitation. False-color images combine information from three photoreceptor channels, with different receptor excitation images plugged into each of the different channels of the RGB screen. Such images provide an impression of the color contrasts that may be visible to each visual system, i.e., *S. barbipes* on the left, and birds on the right. False-color images of both the dichromatic (probable) and trichromatic versions of the *S. barbipes* visual system are shown. To visualize a UM dichromatic visual system, the U photoreceptor was plugged into both the red and blue channels of the computer screen to avoid a strongly tinted image

**Fig. 5:**
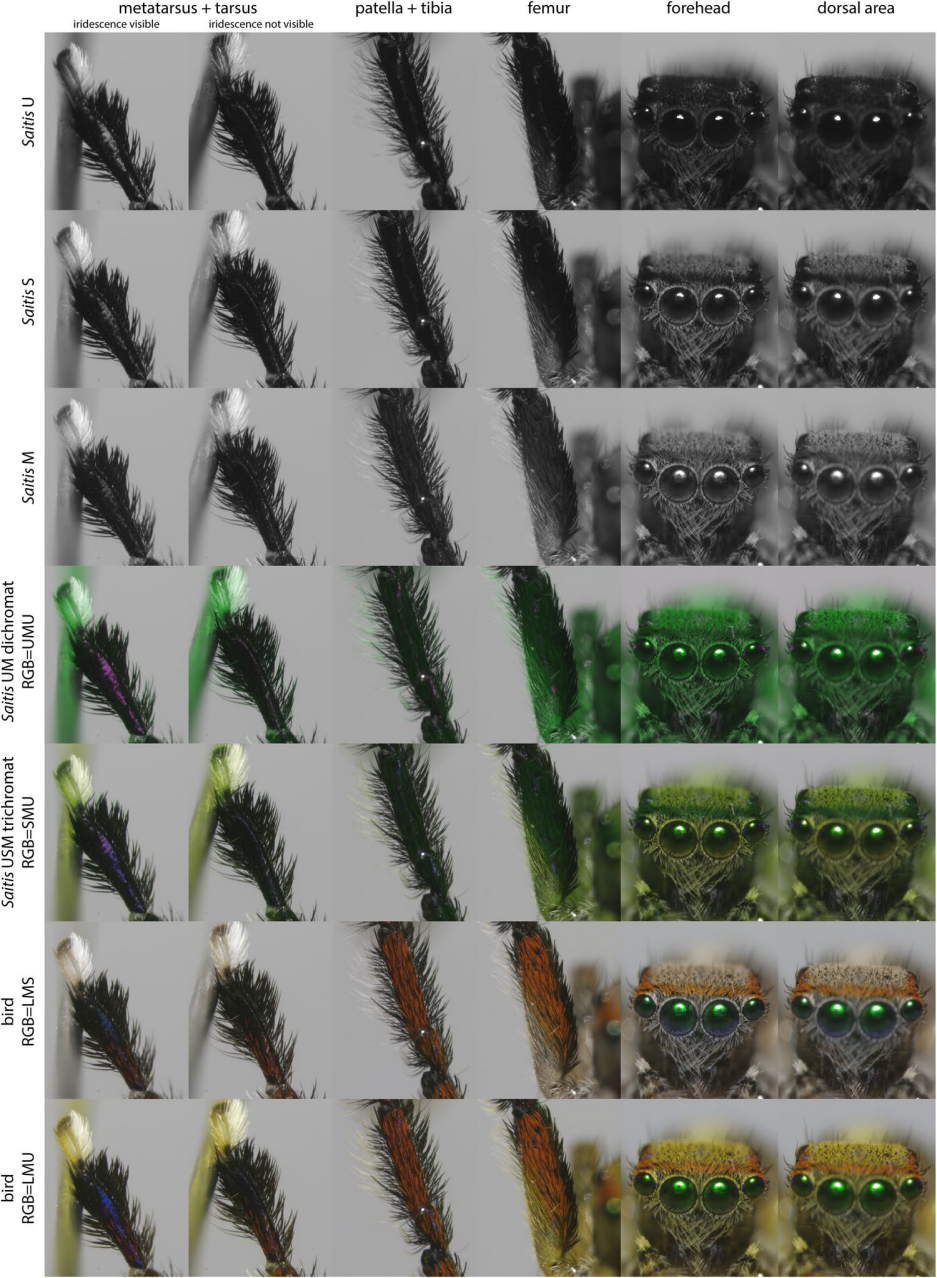
Exemplar images used for color patch selection and analysis. Labels as in Fig. 4. The vertical field of view of each image is 2.2 mm

To get an impression of the spider color pattern as a whole, we took photos of entire spiders posed in a display stance (Fig. 4). To get the spider to fill the whole frame, extension tubes (Kenko Extension Tube Set DG, Kenko Tokina Co., Ltd., Tokyo, Japan) were inserted between the 60 mm lens and the filter wheel of the camera. Photos were taken at different focus depths and then combined in Adobe Photoshop (Adobe Inc., San Jose, CA, USA) using the auto-align and auto-blend functions. To obtain extreme close-up images of individual male body parts for color patch selection and analysis, more extension tubes were added such that individual segments filled the entire frame (Fig. 5). No focus stacking was used in these latter photos. Close-up images were taken of all segments of both the left and right third leg pair. If any part of the spider was found to have been damaged or to have moved slightly between photos taken through different filters, these body parts were excluded from further analysis. Over- and under-exposed pixels were also excluded from analysis.

The camera sensor has a linear response to light, so no non-linearity corrections were needed. Dark noise was obtained from several columns of pixels on the camera sensor that do not receive any light and was subtracted from all pixels that receive the image. Each pixel value of real and computational filter images represented the quantum catch by the simulated photoreceptor class at a single point in space. To adapt quantum catches to the spectral distribution of the illuminant (i.e., convert to relative quantum catches) (Vorobyev et al. 1998), each pixel value was normalized by the mean pixel value of a large selection of the gray standard located behind the spider.

The pixel locations corresponding to the gray standard and color patches of interest on the spider were selected interactively in MATLAB using the “roipoly” function. Nine distinct combinations of colors and structures were identified from avian false-color images and selected on the prosoma and third pair of legs, which are raised and waved during courtship. These included red hair and cuticle, black hair and cuticle, white hair and cuticle, iridescent UV cuticle, orange hair, and beige hair. We used avian false-color images to make selections, since the greatest number of human-discriminable colors could be seen in these photos. For categorization purposes, we use human color names to describe how colors appear in avian visible-light false-color images (i.e., RGB = LMS; see “Methods”: Visualizations and Fig. 5) but acknowledge that these color names reflect what humans see, not animals. As many as possible, up to a maximum of ten, selections of each combination of color and structure (indicated in Table 1) were selected. The position of the fourth segment had to be shifted slightly between shots in order to obtain images in which the UV iridescence was and was not visible.

**Table 1:** Combinations of colors and structures selected on the prosoma and each of the five segments of the third leg pair

		Prosoma		Third leg pair					*N*	
Structure	Color	Forehead	Dorsal	Femur	Patella	Tibia	Metatarsus	Tarsus	Selections	Indivs
Hair	Red	X		X	X	X	X		1938	24
	Orange	X							252	24
	Black					X			360	24
	White							X	357	22
	Beige		X						226	22
Cuticle	Red					X			408	24
	Black						X		401	23
	UV iridescent						X		470	24
	White							X	349	21

It should be noted that *S. barbipes* should only be able to resolve individual hairs at close viewing distances. Even if we assume that *S. barbipes* possesses the best spatial acuity ever reported in a salticid, i.e., the 0.04° interreceptor angle reported in *Portia fimbriata* (Williams and McIntyre 1980), the spider would need to be situated at a viewing distance of ~ 4 mm in order to see individual hairs in sharp focus. At greater distances, the colors of the hair and underlying cuticule would blend together into an intermediate color. In order to estimate how large of an effect the dark cuticular colors surrounding the red, orange, and beige hairs had on the degree of color contrast between these and adjacent black color patches when viewed at greater distances, we additionally selected large, standardized patches on the tibia, metatarsus, forehead, and dorsal prosoma. Color patch boundaries were defined using predetermined landmarks, such as cuticular contours and the edges of eyes. A total of 4761 small patches including only hair or cuticle were selected (Table 1) and a total of 131 large patches including both hair and cuticle were selected.

Pixel locations were saved and later used to calculate the median relative quantum catch *P* of each photoreceptor for each selected color patch. These medians were then converted to non-linear receptor excitation values following Naka and Rushton (1966),

3 $$E=\frac{P}{P+1}$$

Within-channel differences in receptor excitation *E* between color patches were then calculated for each of the spider photoreceptor classes *i*,

4 $$\Delta {E}_{i}={E}_{i, 1}-{E}_{i, 2}$$

Finally, receptor noise-limited (RNL) color contrasts *ΔS* (1) between color patches on the same leg and (2) between color patches on the prosoma and each leg were calculated by plugging *ΔE* values into the RNL color contrast equation (Vorobyev and Osorio 1998; Vorobyev et al. 1998). For trichromats, this was calculated as

5 $$\Delta S=\sqrt{\frac{\omega_1^2{(\Delta E_3-\Delta E_2)}^2+\omega_2^2{(\Delta E_3-\Delta E_1)}^2+\omega_3^2{(\Delta E_1-\Delta E_2)}^2}{{(\omega_1\omega_2)}^2+{(\omega_1\omega_3)}^2+{(\omega_2\omega_3)}^2}},$$

and for dichromats, as.

6 $$\Delta S=\sqrt{\frac{{(\Delta E_1-\Delta E_2)}^2}{\omega_1^2+\omega_2^2},}$$

where *ω*_*i*_ is the standard deviation of the noise in photoreceptor channel *i* and is calculated as

7 $$\omega_i=\frac\upsilon{\sqrt{\eta_i}},$$

where *υ* is the noise in a single photoreceptor and *η*_*i*_ is the relative number of photoreceptors in photoreceptor class *i*. The noise in a single photoreceptor has only been measured in one invertebrate, the honeybee *Apis mellifera*, 0.074 (Vorobyev et al. 2001), which we use as an approximate estimate for salticids. Relative photoreceptor numbers in salticids are also unknown, so we used the relative numbers sampled during microspectrophotometry as an approximation, i.e., 1:7 for the dichromatic ultraviolet:green (U:M) model and 0.4:0.6:7 for the trichromatic ultraviolet:blue:green (U:S:M) model.

### Visualizations

The grayscale images in Figs. 4 and 5 are visualizations of the relative excitation of each photoreceptor class on a pixel-by-pixel basis. To generate such images, we normalized receptor excitations *E*_*i*_ by the maximum receptor excitation value across all color channels of both visual systems (spider and bird). The same values were plugged into each of the R-, G-, and B-channels of the computer display in order to obtain a grayscale image. This gives receptor excitation images in which bright pixels correspond to high excitation and dark pixels to low excitation.

False-color images in Figs. 4 and 5 help one get an approximate sense of the color contrasts that may be visible to different visual systems. Contrasts in the UV most resemble those in the blue, so for tetrachromatic visual systems (i.e., birds), we alternately plugged the S- and U-cone excitation images into the B-channel of the computer display, while keeping the L- and M-cone excitation images plugged into the R- and G-channels of the computer display, respectively. To visualize a trichromatic visual system, each of the different photoreceptor excitation images was plugged into a unique channel of the RGB display. To visualize a dichromatic visual system, one photoreceptor excitation image was plugged into one channel, and the other into two channels, of the RGB display to avoid a strongly tinted image.

## Results

### Microspectrophotometry

Lambda maxes were visualized in a frequency histogram, which revealed the presence of three peaks at UV (U), blue (S), and green (M) wavelengths (Fig. 2a). Linear mixed models fit by restricted maximum likelihood and with individual ID as a random factor revealed no significant differences between male and female peak sensitivities for U (*F*_1,16_ = 2.114, *p* = 0.165), S (*F*_1,3.180_ = 0.353, *p* = 0.592), or M (*F*_1,3.602_ = 6.299, *p* = 0.073) photoreceptor classes. We therefore pooled males and females to calculate non-sex-specific median values (U = 359 nm, S = 451 nm, M = 526 nm). Visual inspection of retinal sections in bright light revealed that *S. barbipes* does not have intraretinal filters.

Jumping spiders have bi-stable photopigments (Varma et al. 2019), and some, if not all, of the measurements with peak absorbance in the blue wavelength range are likely to have been the metarhodopsin state of the ultraviolet-sensitive photopigment. Four pieces of evidence support this possibility. First, in the jumping spider *Salticus scenicus*, we have found that these “blue” spectra are readily photoconverted to spectra characteristic of UV-sensitive visual pigments following exposure to light filtered by a longpass optical glass with transmission above 400 nm (DO & NIM, unpublished results). Second, in *S. barbipes* (and numerous other salticid species, DO & NIM, unpublished results), these “blue” spectra lack clear evidence of a “*cis*” or “*beta*” peak where one would be expected for a blue-sensitive visual pigment, whereas spectra obtained from green-sensitive photoreceptors clearly exhibit such a peak. Third, all “blue” spectra were measured in regions of the retina (i.e., the distal two retinal tiers) where we otherwise find only UV-sensitive photoreceptors in *S. barbipes* and other species. Thus, it is possible that the “blue” spectra represent the metarhodopsin photoproduct of UV-sensitive photoreceptors whose rhodopsins were accidentally photoconverted during bleaching of nearby photoreceptors. Finally, although the ancestral complement of opsins in spiders includes an opsin that should give rise to a blue-sensitive visual pigment (Morehouse et al. 2017), the only conclusive evidence for such a pigment in salticids so far (i.e., in *Hasarius adansoni*) shows this visual pigment to be restricted to the secondary eyes (Terakita and Nagata 2014). Nevertheless, the possibility remains that some of these measurements represent “true” blue-sensitive photoreceptors. Given this uncertainty, the visual modeling was conducted using both possible visual systems (i.e., UV-green (UM) dichromatic and UV-blue-green (USM) trichromatic).

### Lens transmittance

Principal eye lenses from males (5 from 3 individuals) and females (6 from 3 individuals) exhibited high transmittance from 300–700 nm (Fig. 2b). The transmittance of male lenses exhibited a slight dip in the medium- to long-wavelength portion of the spectrum, which likely corresponds to the visible green reflectance of the male cornea. This green reflectance could be seen in both intact and dissected male specimens. The slightly lower transmittance in this portion of the spectrum had a negligible effect on the effective spectral sensitivity of the underlying photoreceptors, however (Fig. 2c).

### Multispectral imaging and visual modeling

**Fig. 6:**
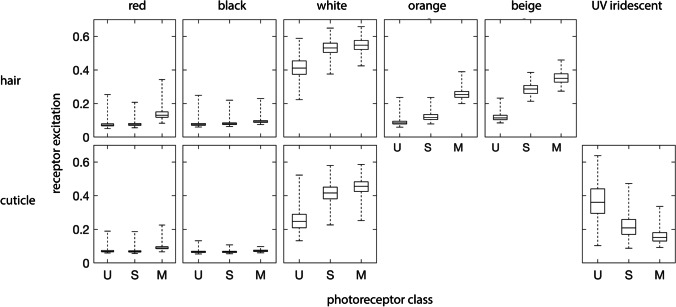
*S. barbipes* photoreceptor excitation values for hair and cuticular color patches

**Fig. 7:**
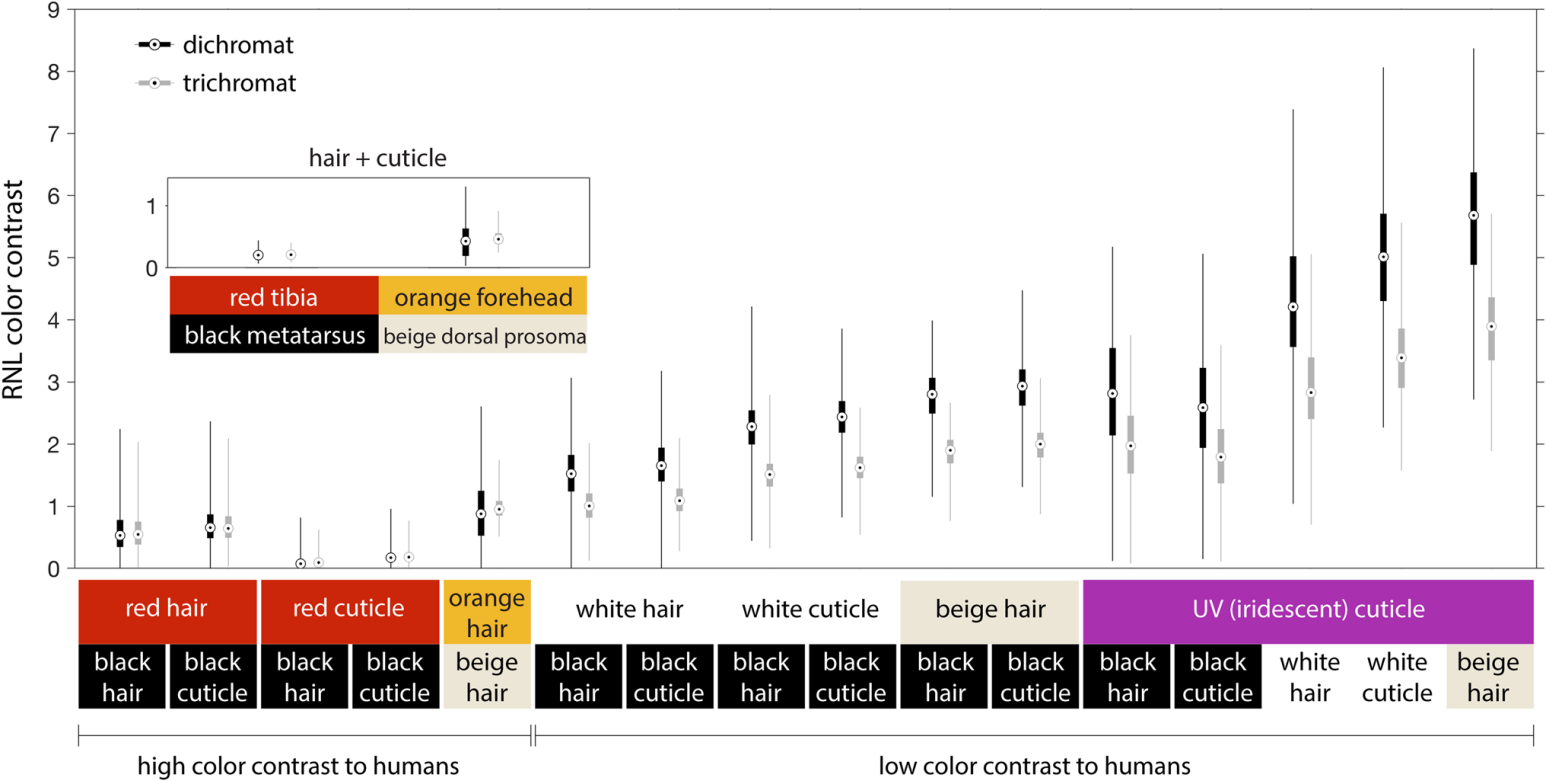
RNL color contrasts between color patches as seen by *S. barbipes* dichromatic (probable) and trichromatic visual systems. The main figure shows contrasts between individual hairs and patches of cuticle, while the inset shows contrasts between larger color patch selections including both hair and underlying cuticle

The relative quantum catches of the different *S. barbipes* photoreceptor classes differed slightly between the red and black color patches, with the M photoreceptor catching more photons from the red color patches than from the black ones (Fig. 6). Color contrast calculations indicated that red–black patch contrast was the lowest source of color contrast across the spider’s body and is likely not perceptible under normal viewing conditions (Fig. 7). If red coloration is perceived as different from black, it would be perceived as a low-luminance green — what one might call a dark “spider-green.” Some regions of the spider’s body that appear bright but nearly achromatic (i.e., beige hair and white cuticle) to human eyes were quite UV-absorbent and, as a result, would be perceived as being a brighter spider-green than the red color patches. Finally, we found an iridescent UV color patch on the metatarsus that contrasted strongly with the spider-green patches dominating the rest of the spider’s coloration. The visibility of this patch was highly angle-dependent, and similar coloration was sometimes visible on small portions of other segments, as, for example, on the tibia in Fig. 4.

## Discussion

Taken together, our results indicate that *S. barbipes* is likely unable to perceive its own red coloration. This is puzzling, especially since its red color patches are concentrated on the forward-facing body surfaces of males. Instead, red and black color patches seem to serve equivalent functions — i.e., in generating strong achromatic contrast with the visual background. Why, then, have *S. barbipes* evolved a combination of red and black coloration, rather than just one or the other?

Red and black coloration may co-exist in *S. barbipes* due to a lack of strong selection for or against either alternative. In spiders, orange, red, and black pigments are commonly ommochromes (Seligy 1972; Insausti and Casas 2008; Riou and Christidès 2010; Hsiung et al. 2017) derived from the amino acid tryptophan (Figon and Casas 2018). Their color is determined by the identity of side chains and/or redox states (Figon and Casas 2018) which likely have similar production costs.

A more functional explanation for red coloration could be that the warm climate of their native range in southern Europe has selected for pigments that reflect light invisible to *S. barbipes* that would otherwise be absorbed and converted to heat. Under this scenario, one might question why *S. barbipes* possesses black coloration at all if red coloration is better for thermoregulation and is perceived equivalently. Selection toward such a phenotype may currently be underway or may be too weak to convert all black coloration to red. It would be interesting to test whether populations living in warmer parts of their range have a greater coverage of red coloration than populations living in cooler parts.

Somewhat counterintuitively, predation pressure from red-sensitive animals, such as birds and lizards, may actually favor a combination of red and black coloration over black coloration alone. Male *S. barbipes* are only about 4 mm in length and, at normal bird and lizard viewing distances, their red and black coloration can be expected to merge into a single brownish or orangish color. Such a color should contrast less with their natural background of leaf litter and rocks than either red or black alone would. The blending of colors at a distance can already be observed by comparing the whole-spider images in Fig. 4 to the close-up photos of individual body parts in Fig. 5. The bright red hairs are more clearly visible in the close-up photos of Fig. 5 because our spatial resolution can better distinguish the individual hairs in these photos. The same may apply to certain insect prey, which, due to optical limitations of compound eyes (Land 1981), will sample *S. barbipes* at a coarse spatial resolution even from a close distance. That said, few arthropods possess red vision (Barth 2002; van der Kooi et al. 2020), and it is more likely that vertebrate predators are the ones exerting natural selection on these colors.

Although we found no evidence of colored filters in the lens or retina, it is possible that there remains an undetected filtering mechanism that shifts the peak sensitivity of *S. barbipes*’ green receptors to red. The salticid retina contains four distinct layers of photoreceptors, with shorter-wavelength-sensitive receptors being located in distal layers and longer-wavelength-sensitive receptors being located in proximal layers (Blest et al. 1981; Nagata et al. 2012). One might hypothesize that absorption of light by distal layers could shift the spectral sensitivity of proximal layers to longer wavelengths. However, following Eq. 3 in Warrant and Nilsson (1998), using a typical invertebrate absorption coefficient of 0.0067 and photoreceptor lengths from related salticids (Blest et al. 1981; Zurek et al. 2015), we calculated that distal layers should only absorb a small proportion of incident light due to their short path length, causing the most proximal layer’s wavelength of peak sensitivity to shift by < 1 nm. It is therefore unlikely that spectral filtering by distal photoreceptors could produce a “hidden” red-sensitive photoreceptor class.

Previous work on other jumping spiders has shown that the salticid dioptric system does not correct for chromatic aberration. This means that longer-wavelength light is focused on proximal retinal layers and shorter-wavelength light is focused on distal retinal layers (Land 1969; Blest et al. 1981; Nagata et al. 2012). In *S. barbipes*, if red light is better focused on the most proximal retinal layer (L1) than on the layer immediately distal to it (L2), the greater concentration of red light on L1 could make it effectively more sensitive to red light than L2, even if the two layers’ opsins have identical spectral sensitivities. That said, L1 would still be stimulated by a large amount of out-of-focus green light, making this a poorly adapted (and possibly ineffectual) mechanism for distinguishing red from green.

Finally, it is possible that our microspectrophotometric examination missed the presence of a red photoreceptor class. This is unlikely given our thorough sampling of all layers of the retina (*N* = 348 measurements) but cannot be completely excluded in the absence of opsin sequence data and immunostaining of the retina.

To conclude, the presence of sexually dimorphic red coloration in a spider that cannot see red is a surprising finding that we cannot yet explain. Future work should focus on testing the possible explanations outlined above.

## Supplementary Information

Below is the link to the electronic supplementary material.

High resolution (TIF 79419 kb)

Supplementary file2 (R 1 KB)

Supplementary file3 (XLSX 15 KB)

Supplementary file4 (XLSX 81 KB)

## Data Availability

Lambda maxes of each measured photoreceptor can be found in Supplementary Dataset 1, and normalized transmittance spectra for each measured spider lens can be found in Supplementary Dataset 2. Multispectral images, color patch selections, and associated metadata are available for download at tedore.net/multispectral.

## Code availability

The R code used to test for sex differences in lambda maxes can be found in Supplementary Code 1. The MATLAB code used to process, analyze, and plot the image data can be found at tedore.net/multispectral.
